# Examining Parameter Invariance in a General Diagnostic Classification Model

**DOI:** 10.3389/fpsyg.2019.02930

**Published:** 2020-01-13

**Authors:** Hamdollah Ravand, Purya Baghaei, Philip Doebler

**Affiliations:** ^1^English Department, Vali-e-Asr University of Rafsanjan, Rafsanjan, Iran; ^2^English Department, Mashhad Branch, Islamic Azad University of Mashhad, Mashhad, Iran; ^3^Department of Statistics, Technical University Dortmund, Dortmund, Germany

**Keywords:** parameter invariance, diagnostic classification models, G-DINA, item response, attribute, reading comprehension

## Abstract

The present study aimed at investigating invariance of a diagnostic classification model (DCM) for reading comprehension across gender. In contrast to models with continuous traits, diagnostic classification models inform mastery of a finite set of latent attributes, e.g., vocabulary or syntax in the reading context, and allow to provide fine grained feedback to learners and instructors. The generalized deterministic, noisy “and” gate (G-DINA) model was fit to item responses of 1000 male and female individuals to a high-stakes reading comprehension test. Use of the G-DINA model allowed for minimal assumption on the relationship of latent attribute profiles and item-specific response probabilities, i.e., the G-DINA model can represent compensatory or non-compensatory relationships of latent attributes and response probabilities. Item parameters were compared across the two samples, and only a small number of item parameters were statistically different between the two groups, corroborating the result of a formal measurement invariance test based on the multigroup G-DINA model. Neither correlations between latent attributes were significantly different across the two groups, nor mastery probabilities for any of the attributes. Model selection at item level showed that from among the 18 items that required multiple attributes, 16 items picked different rules (DCMs) across the groups. While this seems to suggest that the relationship among the attributes of reading comprehension differs across the two groups, a closer inspection of the rules picked by the items showed that almost in all cases the relationships were very similar. If a compensatory DCM was suggested by the G-DINA framework for an item in the female group, a model belonging to the same family resulted for the male group.

## Introduction

An important requirement of construct validity is measurement invariance. Instability of model parameters across different samples of test takers, for example, may call comparability of test results into question and hence its fairness. Structural equation models (SEMs) and item response theory (IRT) have been used routinely to investigate invariance (e.g., Reise et al., 1993; Raju et al., 2002; Meade and Lautenschlager, 2003). Typical SEM models like confirmatory factor analysis or common IRT models like the Rasch model assume that latent ability is continuous. While continuous latent traits are certainly familiar and often useful, some constructs might be more accurately reflected by a finite set of categorical latent attributes. Especially in the context of abilities that are taught formally, the mastery of discrete learning objectives, e.g., with respect to a curriculum or competence model, is a useful alternative formalization. Estimates of latent profiles of attribute mastery resulting from corresponding statistical models are then helpful feedback for learners, their instructors or on a higher level for educational policy (Ravand and Baghaei, 2019).

**FIGURE 1:**
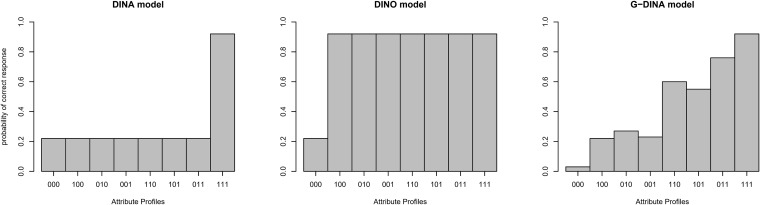
Attribute profiles/Response probabilities for DINA, DINO, and G-DINA for different latent classes (i.e., [000], [100], [010], [001], [110], [101], [011], [111]).

Diagnostic classification models (DCM) are a class of latent trait models that link item level information on the involved attributes to probabilites of correct responses (Rupp and Templin, 2008). To use a DCM to learn about a finite set of latent attributes, a so-called Q-matrix is specified based on substantial expert knowledge, which determines which items are related to which latent attributes (Tatsuoka, 1983). A particular DCM then imposes further structure on the item-specific response probabilities (Tatsuoka, 1985) and is followed up by item parameter estimation. Examples of DCMs include the deterministic noisy “and” gate model (DINA; Junker and Sijtsma, 2001), which assumes that all attributes relevant for an item are necessary for a high response probability, i.e., presence of an attribute cannot compensate for the absence of another. Note that despite its name, the DINA model is probabilistic, i.e., it assumes that persons without a sufficient attribute profile have a chance to guess the item and participants with a perfect profile can still slip, which is reflected in guessing and slipping parameters. The deterministic noisy “or” gate model (DINO; Templin and Henson, 2006) on the other hand is compensatory: If one of the relevant attributes is present, the probability of a correct response is maximal. Another noteworthy DCM and the one used in this work is the generalized deterministic noisy “and” gate (G-DINA) model (de la Torre, 2011), which features a very flexible structure of the relationship of attributes and response probabilities, including (partially) compensatory relationships. This flexibility comes at the price of more model parameters. For a fictional item with three relevant attributes and hence 2^3^ = 8 possible combinations of them, Figure 1 visualizes the response probabilities for DINA, DINO, and G-DINA.

While the DINA model is non-compensatory (all attributes required for a high response probability) in the DINO model mastery of one of the attributes suffices. The G-DINA model is more flexible and contains DINA, DINO, and other models as special cases. In general, the G-DINA model produces partially compensatory response probabilities like the one displayed. Note that the DINA and DINO models feature guessing parameters (a bit above 22% here) and slipping parameters (chance of slipping is around 10% here), while the parametrization in the G-DINA model is more complex. The Additive CDM (ACDM; de la Torre, 2011), linear logistic model (LLM; Maris, 1999), and reduced reparametrized unified model (RRUM; Hartz, 2002) are additive models which are also special cases of the G-DINA. In these models the assumption is that mastery of each attribute contributes to the correct answer regardless of whether the other attributes have been mastered or not. Models such as the DINA, DINO, ACDM, LLM, and RRUM impose one type of relationship (i.e., either compensatory or non-compensatory) on all items of a test while general models such as the G-DINA are flexible enough in that they allow each item to pick a different rule (i.e., model). For example, one item may pick a compensatory model whereas another item might choose a non-compensatory model.

Next to item parameters, DCMs also provide estimates of mastery probabilities for the population at hand, i.e., for each attribute the percentage of individuals having mastered the attribute can be reported. Similarly, covariances of the latent attributes can be studied, which are useful to understand relationships of attributes.

### Invariance Analysis in Foreign Language Testing Research

The invariance of the construct of foreign language ability has previously been tested with multi-group confirmatory factor analysis and differential item functioning (DIF) analysis in item response theory (IRT). Invariance analysis is necessary to ascertain that the test scores have the same meaning across groups and the test is fair for everybody. Muthén (1989) states that “in educational achievement modeling with factor analysis and item response theory, the homogeneity assumption is unrealistic when applied to a sample of students with varying instructional backgrounds” (p. 558).

Invariance analysis in language testing research has been conducted across proficiency levels (Swinton and Powers, 1980; Oltman et al., 1988; Kunnan, 1992; Ginther and Stevens, 1998; Römhild, 2008; Baghaei, 2010), native language (Kim, 2001; Shin, 2005; Forthmann et al., 2019), first language ability, the conditions under which second language is learned (Sang et al., 1986), randomly selected subsamples (In’nami and Koizumi, 2011), across unknown groups using mixed Rasch model (Baghaei and Carstensen, 2013; Aryadoust, 2015; Baghaei et al., 2019) gender (Baghaei et al., 2016), and academic major (Baghaei et al., 2016). Findings of these studies are mixed indicating invariance, partial invariance or non-invariance of second language proficiency across different populations.

### Measurement Invariance in DCMs

Similar to models with continuous latent traits, invariance of item parameters in DCMs is essential: Non-invariance of parameters of DCMs leads to biased classification of individuals across different groups (e.g., male, female). If this is the case, one cannot claim that the test measures the same construct across the groups. Note that similar to the continuous case, invariance is with respect to item parameters: The latent attribute distribution is allowed to vary across groups, so for example females could have higher or lower mastery probabilities than males.

There are few methodological studies (e.g., de la Torre and Lee, 2010; Bradshaw and Madison, 2016) investigating invariance of DCMs. Invariance of model parameters might be impacted by different underlying attribute distributions (e.g., de la Torre and Lee, 2010; Bradshaw and Madison, 2016). Bradshaw and Madison investigated invariance of both person classifications and item parameters under different simulated conditions of sample and test characteristics in the log-linear CDM (LCDM; Henson et al., 2009). They demonstrated that item parameters remained invariant regardless of whether the test was taken by test takers of low, moderate, or high ability. In the same vein, they also found that regardless of the difficulty of the items, test takers’ classifications remained consistent. Bradshaw and Madison found that the invariance property of the LCDM holds when sample size is big enough and the model fits the data. They found that when the assumption of model-data fit is violated, both person and item invariance were impacted. In an earlier study, de la Torre and Lee (2010) examined the invariance of item parameters in the deterministic-input noisy-and-gate (DINA) model (Junker and Sijtsma, 2001). They found that with test takers of low, average, and high abilities item parameters remained consistent given the assumption of model-data fit hold.

However, helpful these methodological studies might be in shedding light on the invariance property of the DCMs, studies with real data are required to check the tenability of the methodological findings in real applications. To this end, the present study aimed at exploring item property invariance of the G-DINA model across male and female groups in a reading comprehension (RC) test. A minor aim of the present study was to compare performance of two gender groups at attribute level.

DCMs provide the means for comparing performances of differing groups of test takers along multiple dimensions besides comparing performance at total score level or via means of continuous latent traits. Total score comparisons provide external estimates of the differences between subgroups performances whereas DCMs offer internal insights into the sources of intergroup differences. Few studies have used DCMs to explore inter-group differences. Tatsuoka et al. (2004) used the Rule Space Method (RSM; Tatsuoka, 1983) to identify the subskills underlying the Third International Math and Science Study-Revised and compared performance of students across a sample of 20 countries at the micro level of the identified attributes. Zhang et al. (2012) compared the civic education achievement of students from three countries across four attributes identified through the General Diagnostic Model (GDM, von Davier, 2005). Chen (2012) compared attribute-level performance of urban and rural Taiwanese students who participated in the Trends in International Mathematics and Science Study (TIMSS). In all the studies mentioned, attribute profiles and mastery probabilities of the subgroups were estimated in separate analyses for each groups and then performances were compared. However, in the present study the multigroup functionality of the CDM R-package (Robitzsch et al., 2018) was used to estimate the attribute profiles of the test takers of male and female groups simultaneously. Furthermore, unlike the previous studies which sufficed to describing the attribute-differences, the present study tested the differences for statistical significance.

The main objective of the present study was to explore invariance with the help of the G-DINA model. Since multigroup G-DINA is a recent development in DCM, we illustrated a step-by-step application of the model. Furthermore, DIF and invariance of other model parameters examined across the two gender groups, have implications for the construct validity of the test under study.

To follow the objectives of the present study, the following research questions were posed:

(1) How stable are item parameters across the gender groups?
(2) Is there any statistically significant difference between skill mastery probabilities for the test takers of the two gender groups?
(3) Do relationships (i.e., compensatory, non-compensatory) among the attributes of RC vary across the two gender groups?

#### Data

Data analyzed in the present study were responses of two equal groups of individuals (500 males and 500 females) who were randomly selected from among the test takers who took the Iranian university entrance examination to enter English programs at master’s level in 2009. For the purpose of the present study, item responses of the selected test takers to the 20 multiple choice RC items were analyzed. It should be noted that guidance of an ethical review board was not sought, as we did not collect the data and analyzed only completely anonymized data provided by the Measurement Organization upon the first author’s formal request. Data of the present study can be made available upon request from the first author.

#### Q-Matrix Development

A key component of every DCM is a Q-matrix which represents attribute-by-item relationships. To define the attributes involved in a test, different sources can be used, including theories of content domain, test specifications, content analysis of the test items, and think-aloud protocol analysis (Leighton et al., 2004; Leighton and Gierl, 2007). In this study we used the data of a non-diagnostic test to extract diagnostic information about test takers’ reading comprehension ability. There was neither test specifications showing what attributes the test items measured nor detailed cognitive model of task performance available.

To determine the attributes that the candidates should have mastered in order to answer the reading comprehension test items, the authors consulted the literature on language ability models in which the reading comprehension attributes and subskills are discussed. The models reviewed included the model proposed by Hughes (2003) consisting of 20 attributes including (1) Identify pronominal references, (2) Identify discourse markers, (3) Interpret complex sentences, (4) Interpret topic sentences, (5) Outline logical organization of a text, (6) Outline the development of an argument, (7) Distinguish general statements from examples, (8) Identify explicitly stated main ideas, (9) Identify implicitly stated main ideas, (10) Recognize writer’s intention, (11) Recognize attitudes and emotions of the writer, (12) Identify addressee or audience for a text, (13) Identify what kind of text is involved (e.g., editorial, diary, etc.), (14) Distinguish fact from opinion, (15) Distinguish hypothesis from fact, (16) Distinguish fact from rumor or hearsay, (17) Infer the meaning of an unknown word from the context, (18) Make propositional informational inferences answering questions beginning with who, when, what, (19) Make propositional explanatory inferences concerned with motivation, cause, consequence and enablement, answering questions beginning with why and how, and (20) Make pragmatic inferences. This model is an instant of a comprehensive model in which all the probable reading attributes are mentioned.

Another model which was consulted in this study is proposed by Farhadi et al. (1994). This model consists of a narrower domain of attributes for reading comprehension. These attributes are: (1) Guess the meaning of words from context, (2) Understand the syntactic structure of the passage, (3) Get explicit and implicit ideas, (4) Grasp the main idea of the passage, (5) Recognize the tone, mood and purpose of the writer, (6) Identify literary techniques of the writer, and (7) Draw inferences about the content of the passage.

Other useful sources for identifying the attributes are the previous research conducted in the field of reading comprehension. Therefore, we referred to numerous studies in which reading comprehension attributes were investigated (Buck et al., 1997; Sheehan, 1997; Jang, 2005; VanderVeen et al., 2007; Lee and Sawaki, 2009; Svetina et al., 2011; Kim, 2015; Ravand, 2016).

Reviewing the existing theories and the RC literature, the first author brainstormed with five expert judges and drew a list of 10 attributes and presented it to the judges. The judges were Iranian university instructors who held PhDs in Applied Linguistics with at least 10 years of teaching and researching RC. Prior to coding the test items, they were trained in a 30-min session on how to code the items for attributes. Then, the 20 RC items and the list of 10 attributes were given to each coder. The judges were asked to read the passages and answer the items and code them independently for each attribute. They were asked to rate how sure they were each attribute was necessary for each item on a scale of one to five. We included in the initial Q-matrix attributes which were rated at least four by at least three of the raters.

In the next step, we subjected the Q-matrix to statistical analysis through the procedure proposed by de la Torre and Chiu (2016) using the GDINA package (Ma et al., 2016) in R. The procedure is based on a discrimination index which measures the degree to which an item discriminates among different reduced q-vectors and can be used in conjunction with the G-DINA and all the constrained models subsumed under it. de la Torre and Chiu’s (2016) procedure identifies potential misspecifications and provides suggestions for modification of the Q-matrix. The suggested modifictions are either turning 0 entries into 1 s or vice versa. Overall, 12 revisions were suggested: In eight cases the suggestion was to turn 0 s into 1 s and in four case it was suggested to turn 1 s into 0 s. The Q-matrix was modified if the suggested changes were theoretically supported. Deletions from the Q-matrix were discussed in a panel discussion with the five expert judges. The final Q-matrix is displayed in Table 1. Vocabulary refers to the ability to identify meaning of words using linguistic and contextual clues and syntax refers to the ability to identify sentence meaning and structure using grammatical and syntactic knowledge. Discourse knowledge refers to knowledge of the meaning of the paragraph or the overall text. Inference refers to the ability to draw a link between textual and contextual clues to understand what is not directly stated. Pragmatic knowledge refers to understanding contextualized implied meaning of a text. For example, contextual, sociolinguistic, sociocultural, psychological, and rhetorical meanings.

**TABLE 1:** Final Q-matrix.

**Item**	**Vocab**	**Syntax**	**Discourse**	**Inference**	**Pragmatic**
1	1	0	1	1	1
2	0	0	1	0	0
3	1	0	1	0	0
4	1	0	1	1	0
5	1	0	1	1	0
6	0	0	1	1	1
7	0	0	1	1	1
8	0	0	1	1	1
9	0	0	1	1	0
10	1	0	1	0	0
11	1	0	0	0	0
12	1	0	0	1	0
13	1	1	0	1	0
14	0	1	1	1	0
15	1	0	1	1	0
16	0	1	1	0	0
17	1	1	0	1	0
18	0	1	1	0	0
19	1	0	1	1	0
20	1	0	1	1	0

#### Analysis

Data were analyzed using the CDM package (Robitzsch et al., 2018) and the GDINA package (Ma and de la Torre, 2018) in R (R Core Team, 2019). The CDM package employs marginal maximum likelihood estimation using the EM algorithm for fitting the models (George et al., 2016).

For the purpose of the present study, the sample was split into two halves based on the gender of the test takers. To answer the first research question, item parameters (i.e., intercepts, main, and interaction effects), tetrachoric correlations between the attributes along with their corresponding jackknife standard errors (SEs) were estimated. Thanks to the estimation of SEs, we were able to test the differences between the groups in terms of statistical significance.

Moreover, the attribute profiles of the two groups were compared. For the purpose of Question 2, the average mastery probabilities for each attribute across the two ability groups were estimated and the differences were tested for statistical significance.

Finally, to answer the last research question, model selection at item level was carried out for the two samples, separately. The models thus selected were compared to investigate stability of the nature of the relationships among the attributes across the two groups. Model selection at item level is a recent development which except for few studies (e.g., Ravand, 2016; Ravand and Robitzsch, 2018) has not been exploited.

To perform multigroup analysis, the following steps were taken:

1. The G-DINA model was fit to the data;
2. Multigroup G-DINA was applied assuming invariance of the model parameters;
3. Multigroup G-DINA was applied assuming non-invariance of the model parameters;
4. The models were compared using a likelihood ratio test;
5. Item parameters, model fit at item level, attribute mastery proportions, tetrachoric correlations between the attributes, and class probabilities across the two gender groups were compared.

## Results

First, a single-group G-DINA model was run. As with any other modeling practice, before interpreting DCM results, model fit has to be investigated. Only after sufficient fit of any given DCM to the data (i.e., absolute fit) has been evidenced, one can proceed to compare the model with other DCMs (i.e., relative fit). There are an array of absolute fit indices generated by the CDM package. However, the issue of fit in DCMs is in its infancy and there are no cut-offs or significance tests for most of these indices. In the present study, MX2 (Chen and Thissen, 1997) which is an averaged difference between model-predicted and observed response frequencies, the mean absolute difference for item-pair correlations (MADcor) statistic (DiBello et al., 2007), the standardized root mean squared residual (SRMSR), and abs(fcor) which is the absolute value of the deviations of Fisher-transformed correlations (Chen et al., 2013) are reported. It should be noted that significance tests are available for MX2 and abs(fcor). Both indices are residual-based and a non-significant value indicates the difference between the model-predicted and observed values are not significantly different from zero, and hence indicate good fit. As the non-significant MX2 and abs(fcor) values in Table 2 show, the G-DINA fits the data. As to SRMSR, Maydeu-Olivares (2013) considered models with values below 0.05 as models with substantively negligible amount of misfit and with regards to MADcor, DiBello et al. (2007) considered a value of 0.049 in Jang (2005) and Roussos et al. (2006a, b) as suggesting a good fit of the DCM to the data. Thus, it seems that the G-DINA fits the data in the present study.

**TABLE 2:** Absolute fit indices.

**Type**	**Value**	***P***
Max(X2)	6.421	1
Abs(fcor)	0.061	0.868
MADcor	0.015	
SRMSR	0.019	

In the next step, a multigroup G-DINA which assumes invariance of item parameters across the groups was to be tested. However, prior to that a differential item functioning (DIF) analysis should be run (George and Robitzsch, 2014). In other words, the assumption that every item functions in the same way in both groups should be tested before estimating a multigroup DCM. In DIF analysis within the framework of DCMs (DCM DIF), probabilities of correct responses to any given item are compared across test takers with the same attribute profiles but from different observed groups (Hou et al., 2014). In other words, in DCM DIF, the matching criterion is the attribute profile of the test takers.

The results of DIF analysis (Table 3) show that for none of the items parameters were significantly different between male and female groups (*p* < 0.05). It should be noted that the last column in Table 3 represents effect sizes for the differences. As small differences might appear to be significantly different when sample size is large, the effect sizes for DCM DIF are also reported. The unassigned area (UA), introduced by Raju (1990) has been adopted as a measure of effect size in DCMs. Jodoin and Gierl (2001) suggest as a rule of thumb values of 0.059 to distinguish negligible from moderate DIF and 0.088 to distinguish moderate from large DIF. The *p*-values obtained from an asymptotic normal assumption were corrected, using the false discovery rate (FDR) correction of Benjamini and Hochberg (1995). This is less conservative than a Holm (1979) correction, which would control the family-wise error. While some of the UA values were larger than 0.059, none of the itemwise tests was significant after a Benjamini-Hochberg FDR correction.

**TABLE 3:** DIF results.

**Item**	**X2**	**df**	**P**	**UA**	**Adusted *p*-values**
1	4.91	16	1.00	0.14	1
2	0.10	2	0.95	0.01	1
3	1.05	4	0.90	0.03	1
4	3.81	8	0.87	0.11	1
5	1.65	8	0.99	0.05	1
6	2.29	8	0.97	0.07	1
7	1.06	8	1.00	0.08	1
8	1.10	8	1.00	0.03	1
9	1.55	4	0.82	0.04	1
10	2.08	4	0.72	0.05	1
11	0.28	2	0.87	0.01	1
12	2.44	4	0.65	0.04	1
13	2.75	8	0.95	0.06	1
14	2.41	8	0.97	0.07	1
15	3.02	8	0.93	0.08	1
16	1.69	4	0.79	0.03	1
17	4.34	8	0.82	0.09	1
18	2.79	4	0.59	0.04	1
19	1.73	8	0.99	0.03	1
20	1.14	8	1.00	0.06	1

In the next step, two multigroup G-DINA models were compared: one with the assumption of invariance (Mod1) and the other with the assumption of non-invariance of item parameters across the two gender groups (Mod2). Note that mean (and covariance) of attribute profiles were group specific in both models. A significant difference in the log-likelihood values indicates that the nested DCM (here Mod2 which is the model with more parameters) fits significantly better. As Table 4 displays, the likelihood ratio test showed a non-significant difference between the two models, so the simpler invariance model is preferred, which is also confirmed in terms of AIC and BIC.

**TABLE 4:** Model comparison.

**Model**	**LL**	**Npars**	**AIC**	**BIC**	**Chi2**	**df**	***P***
Mod1	−21109	164	42546	43451			
Mod2	−21059	296	42711	44345	98.3	132	0.987

*LL, log likelihood value; #Npar, number of parameters; Mod1, multigroup G-DINA with invariance assumption; Mod2, multigroup G-DINA with non-invariance assumption.*

Complementary to the DIF analyses and the global likelihood ratio test item parameter invariance was investigated in detail. Item parameters for the two groups were estimated simultaneously as a multigroup model with the help of the CDM package. Jackknife standard errors were obtained for item parameter differences across the two groups. The jackknife method removes single observations (=response vector) from the dataset one by one, fits the model and averages the calculated estimates. Each Jackknife sample is the original data with a single observation omitted. To estimate the standard errors, 1000 Jackknife samples were used in the present study. In the interest of space, only the parameter differences which were statistically different across male and female groups have been reproduced in Table 5. It should be noted that, overall, 126 parameters were estimated including intercepts, main effects, two-way and three-way interactions. As Table 5 shows, only 10 parameters were significantly different at alpha = 5% between the two groups when ignoring multiple testing issues: six main effects, two two-way interactions, and two three-way interactions. The “value” column in Table 5 represents the difference of the item parameter between males and females and “*t*-value” column is obtained from dividing the values in the “value” column by those in the “jackknife_se”. Without a correction for multiple testing absolute *t*-values of 1.96 and beyond show a significant difference for the respective parameter between the groups, when an asymptotic normal distribution of the *t*-values is appropriate. Notably, most of the non-invariant parameters were main effects which involved Discourse and Pragmatic. However, the 126 *p*-values obtained from an asymptotic normal assumption were corrected, using the false discovery rate (FDR) correction of Benjamini and Hochberg (1995). The last column of Table 5 reports adjusted *p*-values, and as one can see four of the adjusted *p*-values are not significant.

**TABLE 5:** Parameter differences between the two groups.

	**Value**	**Jackknife_se**	***t*-value**	**Adjusted *p*-values**
I1voc.dis.prag	–2.549	1.014	–2.514	0.125
I6dis	1.000	0.063	15.873	0.000
I6inf	1.011	0.290	3.486	0.014
I6prag	1.844	0.122	15.115	0.000
I6dis.prag	–1.772	0.211	–8.398	0.000
I6inf.prag	–1.813	0.496	–3.655	0.013
I7prag	0.565	0.127	4.449	0.003
I8prag	–0.607	0.281	–2.160	0.211
I14syn.dis.inf	–1.245	0.464	–2.683	0.123
I20dis	0.637	0.277	2.300	0.216

*jackknife_se, jackknife standard error; voc, vocabulary; dis, discourse; prag, pragmatic; inf, inference; syn, syntax.*

Patterns of tetrachoric correlations between the attributes are shown in Table 6. We calculated the jackknife standard errors of the differences between attribute correlations across the two groups. As Table 7 shows, none of the correlations are significantly different.

**TABLE 6:** Tetrachoric correlations between the attributes.

	**Vocabulary**	**Syntax**	**Discourse**	**Inference**	**Pragmatic**
Vocabulary	1.000	0.952	0.429	–0.193	0.375
Syntax	0.873	1.000	0.719	–0.305	0.104
Discourse	0.166	0.725	1.000	–0.856	–0.603
Inference	0.710	0.347	–0.404	1.000	0.776
Pragmatic	–0.139	0.790	0.119	–0.132	1.000

*Correlations for females are below the diagonal line and those for males are above the diagonal line.*

**TABLE 7:** Differences between tetrachoric correlations.

**Correlation**	**Value**	**Jackknife_se**	***t*-value**
voc-syn	–0.079	0.098	–0.806
voc-dis	–0.263	0.427	–0.616
voc-inf	0.904	1.004	0.900
voc-prag	–0.514	1.450	–0.354
syn-dis	0.006	0.275	0.022
syn-inf	0.653	1.088	0.600
syn-prag	0.686	0.750	0.915
dis-inf	0.452	0.675	0.670
dis-prag	0.722	1.111	0.650
inf-prag	–0.908	0.974	–0.932

*jackknife_se, jackknife standard error; voc, vocabulary; syn, syntax; dis, discourse; inf, inference; prag, pragmatic.*

To further investigate the group differences, the average probabilities of attribute mastery across the two groups were compared. As Table 8 shows, attribute mastery probabilities are very similar in both groups. Vocabulary and inference were the easiest and the most difficult attributes for both groups, respectively. The second easiest skill for both groups was syntax. The Jackknife method was used to estimate standard error of the differences between skill possessions across the two groups. As Table 8 shows, there were no significant statistical differences in skill possessions across male and female groups.

**TABLE 8:** Attribute mastery probabilities.

	**Female**	**Male**	**Value**	**jacknife_se**	***t*-value**
Pragmatic	0.477	0.524	–0.043	0.099	–0.434
Inference	0.343	0.367	–0.027	0.100	–0.270
Discourse	0.484	0.406	0.073	0.215	0.339
Syntax	0.511	0.712	–0.197	0.271	–0.726
Vocab	0.689	0.734	–0.048	0.608	–0.078

*Jkse, jackknife standard error.*

**FIGURE 2:**
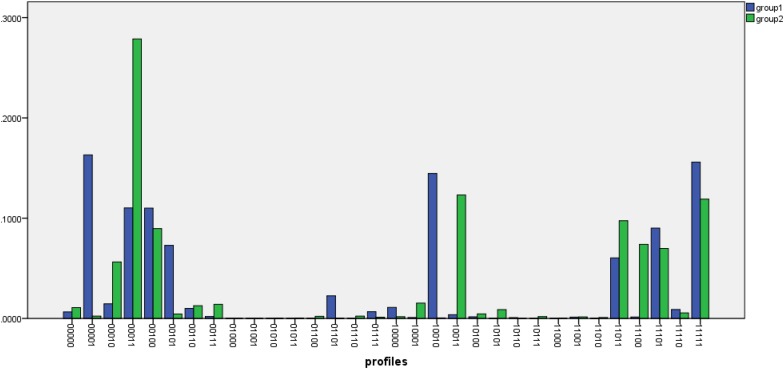
Probabilities of the attribute profiles across females (group1) and male (group2).

In order to compare performance of the two groups, we further compared the attribute profiles of the test takers across the male and female samples. Figure 2 shows the differences between the class probabilities for the two groups. Attribute profiles with probabilities of at least 0.1 where the differences between the male and female groups are sharp has also been displayed in Table 9. For the female group, the three most prevalent attributes profiles were [10010], [00011], and [00001], into which about 14, 11, and 16% of the respondents, respectively, were classified.

**TABLE 9:** Selected class probabilities across gender.

**Class**	**Females**	**Males**
10010	0.145	0.001
00011	0.110	0.279
00001	0.163	0.002
10011	0.004	0.123

For the male group, there were two attribute profiles whose membership was about 10% and higher as follows: [00011] and [10011] populated by about 28 and 12%, respectively. A comparison of the attribute profiles across the two groups shows that the attribute profile [00011] was among the highly prevalent attribute profiles in both groups. Another commonality between the groups was that the two flat attribute profiles of [00000] and [11111] were not prevalent in either male or female groups. A notable pattern in Table 9 is that about 40% of the respondents in the male group (about 28 and 12% in the attribute profiles [00011] and [10011], respectively) have mastered Inference and Pragmatic.

We also examined the relationships among the attributes at item level across the groups. To this end, model selection was carried out at item level (see Ravand, 2016), separately for the two groups. The results (Table 10) showed that from among the 18 items that required multiple attributes, 13 items picked different rules (i.e., compensatory/non-compensatory models) across the two groups.

**TABLE 10:** Rules picked by items across the groups.

	**Females**	**Males**
Item1	LLM	LLM
Item3	LLM	DINA
Item4	RRUM	LLM
Item5	RRUM	LLM
Item6	LLM	RRUM
Item7	ACDM	LLM
Item8	RRUM	LLM
Item9	LLM	ACDM
Item10	DINO	LLM
Item12	ACDM	ACDM
Item13	RRUM	DINO
Item14	DINO	RRUM
Item15	LLM	LLM
Item16	DINO	DINO
Item17	LLM	DINA
Item18	LLM	RRUM
Item19	LLM	RRUM
Item20	LLM	LLM

*DINA, deterministic noisy Input-and- gate; DINO, deterministic noisy input-or- gate; RRUM, reduced reparametrized unified model; LLM, linear logistic model; ACDM, additive cognitive diagnostic model.*

## Discussion

The present study investigated parameter invariance of the G-DINA model across males and female participants of a high stakes reading comprehension test. First, five attributes underlying performance were identified through qualitative and empirical analysis. As a prerequisite for multigroup G-DINA analysis, a DIF analysis was performed which showed that no items were functioning differentially across the two groups. In the next step, two multigroup models, one with invariant and the other with non-invariant item parameter assumptions, were compared. The likelihood ratio test and model fit indices were consistent with an invariance assumption. The likelihood ratio test was non-significant, indicating that employing the additional parameters of the non-invariant model and hence increasing the model’s complexity was not supported by the data. Item parameters were also compared across the two samples. The results showed that from among 126 intercepts, main effects, two- and three-way interaction effects about 8% parameters were significantly different across the two samples, prior to correction for multiple testing. However, from among the originally 10 parameters with significant differences only six were found to be significant using the adjusted *p*-values. The better fit of the model with invariant item parameter assumption was corroborated by the fact that a relatively small number of item parameters were significantly different between the two groups. One possible reason for the significant differences mentioned might be the sample size used in the present study. Previous studies (e.g., Bradshaw and Madison, 2016) have shown that the invariance property of the DCMs holds when sample size is big enough and the model fits the data. Although the G-DINA used in the present study fits the data, the sample size (i.e., about 500 per group) was relatively small, compared to the DCM studies in the literature. Generally speaking, evidence for the invariance of a model in a population is provided when model fit under invariance constraints is acceptable in a reasonably large sample, e.g., in terms of a model fit index. The same model might be non-invariant in a sample from a different population. The results also showed that correlations between the attributes were not significantly different across the two groups. Overall, the results suggest that the G-DINA is partially invariant across the male and female groups.

The results showed that despite some differences of the mastery probabilities for all the attributes, mastery probabilities for none of the attributes were statistically different across the two groups. Syntax and Vocabulary were the first and second easiest attributes for both group whereas Inference and Pragmatic were the first and second most difficult attributes for both groups. The order of the difficulty of the attributes is aligned with the common sense belief that vocabulary and syntax should be mastered before the mastery of attributes such as inference. However, the pattern of the most prevalent attribute profiles for the male group suggests that Inference and Pragmatic are attributes whose mastery is most probably a prerequisite to the mastery of the other subskills, as indicated by the prevalence of the attribute profiles [00011] and [10011].

Model selection at item level showed that from among the 18 items that required multiple attributes, 13 items picked different DCMs across the groups. On the face of it, it may seem that the relationship among the attributes of reading comprehension differs diametrically across the two groups. Nevertheless, a closer inspection of the rules picked by the items shows that almost in all cases the relationships were very similar: If an item in the female group picked a compensatory rule, it picked a rule belonging to the same family of rules in the other group. It should be noted that LLM, ACDM, DINO, and RRUM are all compensatory DCMs. For more information on categorization of DCMs see Ravand and Baghaei (2019).

In a nutshell, the G-DINA showed invariance across the two groups. There were some significant differences in item parameters across the groups. Future studies can explore invariance of the G-DINA using larger sample sizes. The present study only investigated invariance of item parameters, future studies may use items of differing difficulty levels to also explore person classification invariance. However, conducting such a study entails developing a diagnostic test from the scratch, rather than retrofitting DCMs to an already-existing non-diagnostic test. Currently most of the DCM studies are of retrofitting type (Ravand and Baghaei, 2019).

As to the performance of the two groups, the results showed that there were differences in their attribute profile memberships. However, in terms of the average skill possession across the two groups, there were no statistically significant differences in two of the five skills underlying the reading comprehension test under study. Finally, in terms of the stability of the relationships among the attributes across the two groups, the results showed that the relationships remained invariant in most multi-attribute items. It can be concluded that the models parameters are comparable across the male and female groups.

## Data Availability Statement

The datasets analyzed in this article are not publicly available. Requests to access the datasets should be directed to HR (ravand@vru.ac.ir).

## Author Contributions

HR came up with the idea, wrote the sections “Introduction” and “Discussion,” and did the analyses. PB wrote the review and helped to write the section “Introduction.” PD wrote the review, helped to write the section “Introduction,” and helped with the analyses and the R codes.

## Conflict of Interest

The authors declare that the research was conducted in the absence of any commercial or financial relationships that could be construed as a potential conflict of interest.
